# Establishment of a blocking enzyme-linked immunosorbent assay based on tandem expression of dominant antigenic epitopes of the nucleocapsid protein from attenuated and virulent peste des petits ruminants virus

**DOI:** 10.3389/fcimb.2026.1841234

**Published:** 2026-06-03

**Authors:** Kaiyu Zhu, Xiaolong Gao, Lina Tong, Xinchao Du, Yun Chen, Jinxin Xie, Shanhui Ren

**Affiliations:** 1 College of Agriculture and Animal Husbandry, Qinghai University, Xining, Qinghai, China; 2 College of Veterinary Medicine, Xinjiang Agricultural University, Urumqi, China; 3 State Key Laboratory for Animal Disease Control and Prevention, Lanzhou Veterinary Research Institute, Chinese Academy of Agricultural Sciences, Lanzhou, China

**Keywords:** blocking enzyme-linked immunosorbent assay, monoclonal antibody, nucleocapsid protein, peste des petits ruminants, serological detection, tandem dominant epitope

## Abstract

Peste des petits ruminants (PPR) is an acute, contagious disease caused by the Peste des Petits Ruminants virus (PPRV), mainly affecting small ruminants such as goats and sheep. PPR poses a serious threat to the healthy development of the livestock industry and wildlife safety. To develop a rapid method for detecting antibodies against the PPRV N protein, we analyzed the dominant antigenic epitopes of the N protein of virulent and attenuated PPRV strains using bioinformatics. The dominant antigenic epitopes of the N gene were linked in tandem via overlapping PCR and cloned into a prokaryotic expression system, yielding the recombinant plasmid, pET28a-PPRV-2N (encoding amino acids 1–250). Under optimized expression conditions, recombinant protein 2N was successfully produced after induction with isopropyl β-D-1-thiogalactopyranoside and purified. Using the purified recombinant PPRV-2N as the coating antigen in an enzyme-linked immunosorbent assay, the concentrations of the coating antigen and HRP-labeled monoclonal antibody against the PPRV N protein were optimized, resulting in the successful development of a blocking enzyme-linked immunosorbent assay (ELISA) for PPRV antibody detection. The new blocking ELISA detected positive serum at a minimum dilution of 1:10. The intra- and inter-assay coefficients of variation (CV) were both below 15%. No cross-reactivity was observed with sera positive for other important pathogens. When applied to 100 clinical serum samples, this method showed a 93% concordance with a commercial detection kit. The developed blocking ELISA method demonstrated good specificity, sensitivity, and repeatability, making it suitable for detecting PPRV antibodies. This technique supports the evaluation of PPRV vaccine-induced immune efficacy and aids in disease prevention and control.

## 1 Introduction

Peste des petits ruminants (PPR) is an acute, severe, and highly contagious disease caused by the Peste des petits ruminants virus (PPRV). PPRV primarily infects small ruminants such as goats and sheep, and several wild artiodactyl species are vulnerable hosts (Munir, 2014; Schulz et al., 2018). The clinical signs of PPRV-infected ruminants include stomatitis, fever, and diarrhea, with high rates of morbidity and mortality (Pope et al., 2013). PPR was first identified in Côte d’Ivoire, West Africa, in 1942 and subsequently spread to several countries across the Sahelian–equatorial transition zone (Gargadennec and Lalanne, 1942). The World Organization for Animal Health (WOAH) lists PPR as a notifiable disease and categorizes it as a Category I animal disease in China (Liu et al., 2018). To reduce the serious impact of PPR, the WOAH and the Food and Agriculture Organization of the United Nations (FAO) have jointly developed a strategy to eradicate the disease worldwide by 2030. Currently, there is no effective treatment for PPR; therefore, prevention relies mainly on vaccination with weakened PPRV strains, such as the Lineage II strain, Nigeria 75/1, and vaccines based on Lineage IV strains, including Sungri/96, Arasur/87, and Coimbatore/97 (Sen et al., 2010; Diallo et al., 1989). However, in real clinical practice, challenges such as improper vaccine storage and transport during immunization, inadequate vaccination procedures, high outdoor temperatures, and inter-animal variations often lead to inconsistent antibody levels in vaccinated herds and inadequate vaccination coverage, making PPR prevention and control more complex and challenging.

PPRV is a non-segmented, negative-sense single-stranded RNA virus with a genome of approximately 15,948 nucleotides. In the 3′-to-5′ direction, the genome encodes six structural proteins: nucleocapsid (N), phosphoprotein (P), matrix (M), fusion (F), hemagglutinin-neuraminidase (HN), and large polymerase (L). The P gene also encodes two non-structural proteins (C and V) via alternative start codon usage and RNA editing, respectively (Mahapatra et al., 2003). The N protein is the most abundant and highly conserved viral protein in PPRV-infected cells. It encapsulates the viral genome to form the nucleocapsid and assembles with the P and L proteins to create the replication-transcription complex (RNP), playing a key role in viral genome replication and transcription (Parida et al., 2015; Bhella et al., 2002; Zhu et al., 2019). The N protein also inhibits the production of type I interferon (IFN) by competitively binding to the host transcription factor interferon regulatory factor 3, thus blocking IFN signaling and exerting an interferon-antagonistic function (Zhu et al., 2019). Additionally, the dynamic regulation of stress granules mediated by N protein is essential for efficient PPRV replication (Chen et al., 2023). Owing to its high expression during viral infection, strong immunogenicity, and relatively conserved coding sequence, the PPRV N protein is an ideal target for developing diagnostic antigens and detection methods. Effective epidemic surveillance and evaluation of vaccine immune efficacy depend on accurate, sensitive, and specific serological detection methods for PPRV. Currently, virus neutralization tests (VNT), hemagglutination inhibition assays (HI), counter immunoelectrophoresis (CIE), indirect enzyme-linked immunosorbent assays (iELISA), and competitive ELISAs are used for PPRV antibody detection. However, some of these methods are limited by complex procedures, are highly time-consuming, or require specialized equipment. Therefore, developing a rapid, simple, and reliable detection method is important for large-scale clinical screening and on-site surveillance in primary settings.

In this study, bioinformatics was used to analyze the dominant antigenic regions of the N gene in virulent and attenuated PPRV strains. The N gene sequences from attenuated and virulent PPRV strains were linked via overlapping PCR and cloned into a prokaryotic expression vector for expression and purification. The purified recombinant N protein was then paired with an N protein-specific monoclonal antibody to develop a blocking ELISA for the detection of antibodies against the PPRV N protein. The established method demonstrated good reactivity, specificity, and sensitivity, providing an effective technical approach for PPRV antibody detection, epidemiological studies, and disease prevention and control.

## 2 Materials and methods

### 2.1 Main reagents

The Gel DNA Recovery and plasmid mini-preparation kits were purchased from OMEGA Bio-Tek, Inc. (USA). Restriction endonucleases *BamH* I-HF and *EcoR* I-HF were purchased from New England Biolabs (Ipswich MA, USA). T4 DNA ligase and PCR premix were purchased from Vazyme Biotech Co., Ltd. (Nanjing, China). The PPR competitive ELISA antibody detection kit was purchased from Lanzhou Veterinary Research Institute Biotechnology Co., Ltd. DMEM medium was purchased from Gibco (Lanzhou, China). Freund’s complete and incomplete adjuvants were purchased from Thermo Fisher Scientific Biotechnology Co., Ltd. (Shanghai, China).

### 2.2 Cells, recombinant plasmids, sera, and experimental animals

Mouse myeloma SP2/0 cells, eukaryotic expression plasmids for virulent PPRV N (pCAGGS-virulent PPRV-N-Flag) and attenuated PPRV N (pCAGGS-attenuated PPRV-N-Flag), prokaryotic expression plasmid pET28a, PPRV-negative serum, as well as positive sera against goatpox virus (GTPV), lumpy skin disease virus (LSDV), orf virus (ORFV), and PPRV were preserved by the Lanzhou Veterinary Research Institute, Chinese Academy of Agricultural Sciences. The His-tag antibody was purchased from ABclonal Biotech Co., Ltd. (Wuhan, China). One hundred clinical serum samples were collected from sheep farms in Gansu and Qinghai Provinces. Ten healthy 10-week-old BALB/c mice were obtained from the Lanzhou Veterinary Research Institute of the Chinese Academy of Agricultural Sciences, Lanzhou, China.

### 2.3 Construction and induced expression of prokaryotic-expressed PPRV 2N protein

PCR primers were designed based on the N gene sequences of virulent and attenuated PPRV strains. The major antigenic regions of the two N genes were fused in tandem via overlapping PCR. The gel-purified PCR products were then cloned into the prokaryotic expression plasmid pET28a via homologous recombination, and the recombinant plasmid was named pET-28a-PPRV-2N. The constructed plasmid was transformed into E. coli Rosetta competent cells. A single colony was inoculated into 2 YT medium containing kanamycin and incubated at 37 °C with shaking for 12 h to generate the seed culture. The seed culture was then diluted 1:100 (v/v) in 2 YT liquid medium containing kanamycin and incubated at 37 °C with shaking at 220 rpm until the OD_600_ reached approximately 0.6. IPTG was added to a final concentration of 0.5 mmol/L to induce protein expression, and incubation was continued at 37 °C with shaking at 220 rpm for 12 h. A 1 mL sample of the bacterial culture was centrifuged at 12,000 rpm for 3 min to pellet the cells, and the supernatant was then discarded. The cell pellets were resuspended in 600 μL of PBS (0.01 M, pH 7.4), washed once by centrifugation, and finally resuspended in 100 μL PBS. An equal volume of 2× SDS-PAGE loading buffer was added, the mixture was thoroughly vortexed, and then heated at 100 °C for 15 min in a metal bath. Protein expression was analyzed by SDS-PAGE.

### 2.4 Large-scale induced expression and purification of recombinant PPRV-2N protein

After optimizing the induction conditions, the recombinant protein was expressed and purified on a large scale using the optimized conditions. The seed culture was inoculated into 2 YT medium and incubated for 3 h. Then, IPTG was added to a final concentration of 1 mmol/L (optimal condition), and induction was carried out at 30 °C for 14 h. The bacterial cells were harvested by centrifugation, and the pellet was washed three times with 0.01 mol/L PBS (pH 7.4). TE buffer was added at a 1:80 (w/v) ratio, based on the wet weight of the cells. The mixture was thoroughly resuspended, and the cells were disrupted via ultrasonication. The inclusion body pellet was collected and washed with washing buffer I at a 1:50 (w/v) ratio, with stirring at 200 rpm for 2 h at room temperature. After centrifugation at 6,080 × g for 40 min at 4 °C, the pellet was collected and washed once. Next, the pellet was washed with washing buffer II under the same conditions and dissolved in a denaturation buffer containing 1% N-lauroyl sarcosine. The mixture was stirred overnight at 120 rpm at room temperature, centrifuged at 6,080 × g for 30 min at 25 °C, and the supernatant was collected as denatured inclusion bodies. The samples were placed in a dialysis bag and dialyzed against refolding buffers I, II, III, and IV at a 1:100 volume ratio (denatured sample: refolding buffer). Dialysis was performed at 4 °C under gentle stirring for 12 h in each buffer solution. Afterward, the refolded protein was analyzed by SDS-PAGE, and its reactivity with PPRV-positive serum was tested by western blotting. The target protein was further confirmed using a tag-specific antibody.

### 2.5 Preparation and identification of N protein monoclonal antibodies

Eight-week-old female BALB/c mice were immunized subcutaneously at multiple sites with the purified N protein emulsified in an equal volume of Freund’s complete adjuvant, and 100 μL per mouse was administered for the primary immunization. Two additional booster immunizations were administered at two-week intervals using N protein emulsified in Freund’s incomplete adjuvant. Two weeks after the third immunization, blood samples were collected, and the sera were separated. Antibody titers were measured using indirect ELISA, and mice with high serum titers were selected for the final booster immunization. Three days later, splenocytes from immunized mice were harvested and fused with SP2/0 myeloma cells. Positive hybridomas were screened using indirect ELISA and immunofluorescence assay. These hybridoma cells were subjected to three rounds of subcloning by limiting dilution until a 100% positive rate was achieved. The resulting hybridoma cell lines were expanded, and ascitic fluid containing the monoclonal antibodies was prepared. Monoclonal antibodies were purified from ascitic fluid using caprylic acid–saturated ammonium sulfate precipitation. The purified monoclonal antibodies were characterized by indirect ELISA and labeled with horseradish peroxidase (HRP) using an HRP-labeling kit.

### 2.6 Determination of optimal reaction conditions for indirect ELISA

The checkerboard titration method was used to optimize the reaction conditions. The purified N protein was diluted to 10, 5, 4, 3, 2, 1, 0.75, 0.5, 0.25, and 0.1 ng/mL. Each concentration was added to the ELISA plates at 100 μL per well in vertical columns, with concentrations decreasing sequentially. The plates were then incubated overnight at 4 °C. After blocking with 1% BSA at 37 °C for 1 h and washing three times with PBST, the HRP-labeled monoclonal antibody was serially diluted at ratios of 1:500, 1:1000, 1:2000, and 1:4000. The diluted antibody was added horizontally to the plates at 100 μL per well, with each dilution applied to two adjacent rows. The plates were incubated at 37 °C for 40 min. After washing three times with PBST, TMB substrate solution was added, and the plates were incubated in the dark for 15 min to develop color. The reaction was stopped by adding a stop solution, and OD_450_ values were measured. A combination with an OD_450_ value of approximately 2.0 was chosen as the optimal dilution condition.

Based on the optimized conditions, after blocking, positive and negative control sera were selected and serially diluted in both undiluted form and at ratios of 1:1, 1:2, 1:4, 1:10, 1:20, 1:40, and 1:80. The diluted sera were added horizontally to the wells at 100 μL per well, with one row designated for positive serum and one for negative serum at each dilution. The plates were incubated at room temperature for 1 h, and then the HRP-labeled monoclonal antibody was added. The optimal serum dilution was determined by calculating the P/N ratio for each dilution. Subsequently, additional conditions were optimized, including the blocking buffer (1%, 5%, and 10% BSA and 5% non-fat milk), serum incubation time (30, 45, and 60 min), and substrate development time (10, 15, and 20 min). The optimal reaction conditions for the blocking ELISA were selected to achieve an OD_450_ of approximately 1.5 for the negative control while minimizing the P/N ratio (the ratio of the positive control OD_450_ to the negative control OD_450_).

### 2.7 Determination of the cut-off value for the blocking ELISA method

Forty PPRV-negative serum samples were selected using the PPRV antibody competitive ELISA method recommended by WOAH. These samples were tested using the optimized blocking ELISA method established in this study. The percent inhibition (PI) was calculated using the following formula: PI = [(OD_450_ value of negative control serum − OD_450_ value of test serum)/OD_450_ value of negative control serum] × 100%. The mean PI value and standard deviation (SD) were calculated. The cutoff value was set to the mean PI plus three standard deviations (SDs) (PI mean + 3 SD), establishing the threshold for the blocking ELISA method.

### 2.8 Validation of specificity, sensitivity, and repeatability for the blocking ELISA

To evaluate the specificity of the optimized blocking ELISA method, LSDV-, GTPV-, ORFV-, and PPRV-positive sera, as well as PPRV-negative serum, were tested using this method. Each serum sample was tested in triplicate. To compare the sensitivity of the established blocking ELISA method with that of the competitive ELISA kit manufactured by Lanzhou Veterinary Research Bio-Technology Co., Ltd., the PPRV antibody-positive control was subjected to twofold serial dilutions (from 1:10 to 1:160) and tested using both methods. To assess the intra- and inter-assay repeatability of the method, four serum samples were tested using microplates coated with the same and different batches. Each sample was tested in quadruplicate with two plates tested in parallel. The coefficient of variation (CV) was calculated for each condition tested.

### 2.9 Detection of clinical samples using blocking ELISA

The optimized blocking ELISA method and a commercial PPR competitive ELISA antibody detection kit were used to test 100 clinical serum samples. The results of the two methods were compared, and the concordance rate of the established blocking ELISA method was calculated.

## 3 Results

### 3.1 Expression and validation of the induced-PPRV 2N protein

**Figure 1:**
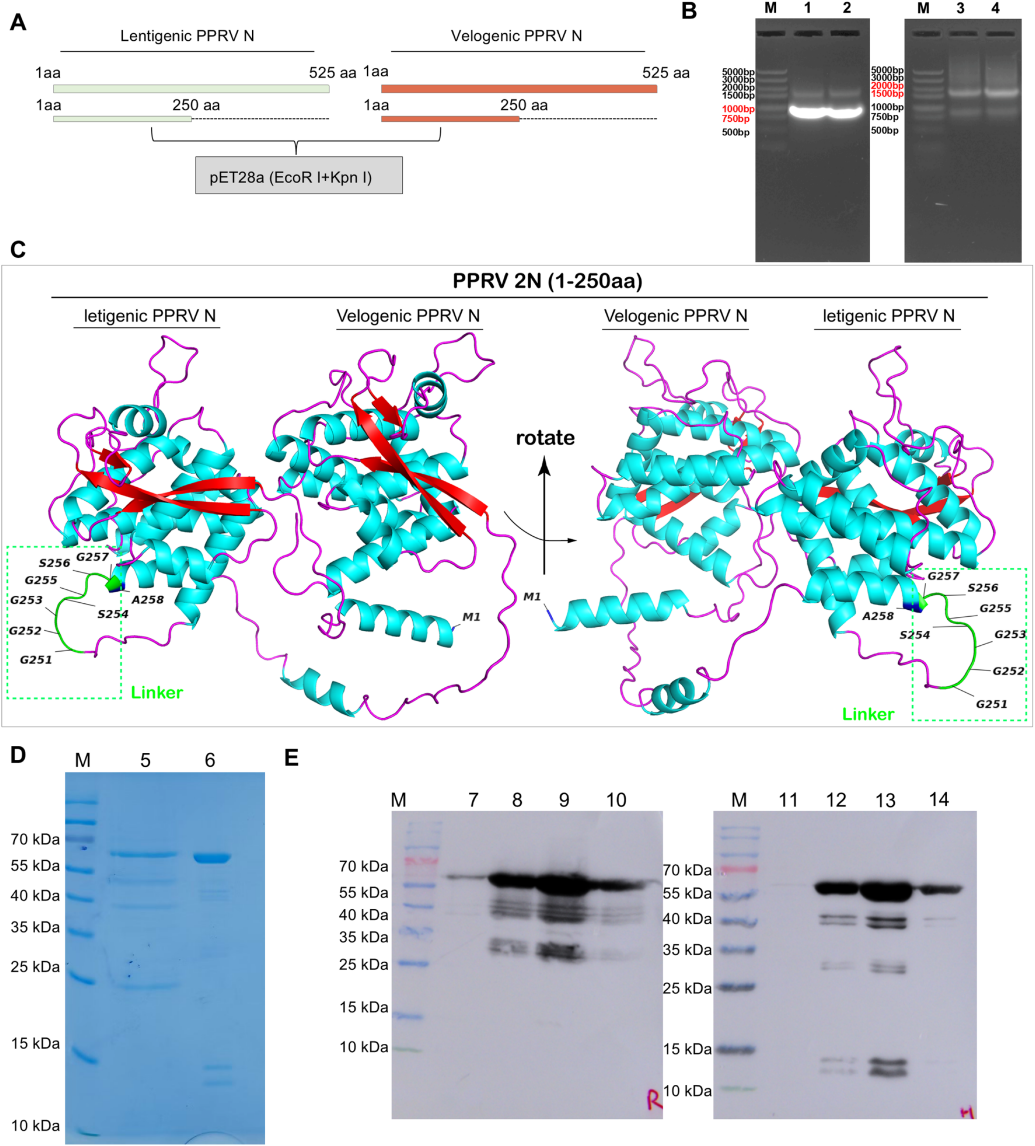
Expression and identification results of the PPRV-N protein. **(A)** Schematic representation of the ligation sequences of pCAGGS-virulent PPRV-N-Flag, pCAGGS-attenuated PPRV-N-Flag, and pET28a plasmid. **(B)** Tandem ligation results of virulent/attenuated N genes. Lane 1: Amplification product of the virulent N protein coding gene. Lane 2: Amplification product of the attenuated N protein coding gene. Lanes 3 and 4: Overlapping PCR amplification products of the virulent and attenuated N genes, respectively. **(C)** Structural prediction diagram of the recombinant PPRV 2N protein. **(D)** SDS-PAGE analysis of the PPRV-N protein before and after purification. Protein molecular weight markers. Lane 5: Protein sample before dialysis purification. Lane 6: Protein sample after dialysis purification. **(E)** Western blot analysis of the purified PPRV-N protein. The left panel shows rabbit anti-goat serum as the secondary antibody, and the right panel shows a His-tag antibody. Lanes 7 and 11: MOCK protein samples. Lanes 8 and 12: 10 μL purified protein samples. Lanes 9 and 13: 20 μL of purified protein samples; lanes 10 and 14: 5 μL of purified protein samples.

To construct a prokaryotic expression vector for the tandem expression of the dominant epitope of the PPRV N protein (**Figure 1A**, **Supplementary Figure 1**), the dominant epitope-coding genes of the N protein from both virulent and attenuated strains, each approximately 800 bp long, were successfully amplified using conventional PCR. The results are shown in **Figure 1B**. After gel extraction of these epitope-coding genes from the virulent and attenuated strains, tandem virulent/attenuated N genes with a total length of 1600 bp were obtained through overlap PCR (**Figure 1B**, lanes 3 and 4). Next, the tandem PPRV 2N gene was cloned into the pET-28a vector via homologous recombination to produce pET-28a-PPRV-2N plasmid. **Figure 1C**, **Supplementary Figure 1C** show a schematic illustration of the structural model of the PPRV-2N recombinant protein connected by a linker (GGGSGSG).

To further confirm that the prokaryotic expression vector could express the 2N protein, the pET-28a-PPRV-2N plasmid was transformed into Rosetta competent cells and induced for protein expression. The expressed protein was purified via dialysis and analyzed using SDS-PAGE and Western blot. As shown in **Figure 1D**, a single band appeared at 55–70 kDa, which matched the expected size. To further validate the expressed protein, Western blot analysis was performed using PPRV-positive serum as the primary antibody and an HRP-labeled rabbit anti-goat antibody as the secondary antibody. Under the same conditions, a His-tag antibody was used as the secondary antibody to confirm the target protein. As shown in **Figure 1E**, the purified recombinant protein strongly reacted with PPRV-positive serum and the His-tag antibody, suggesting successful expression of the tandem PPRV-2N protein.

### 3.2 Establishment of the blocking ELISA method

**Figure 2:**
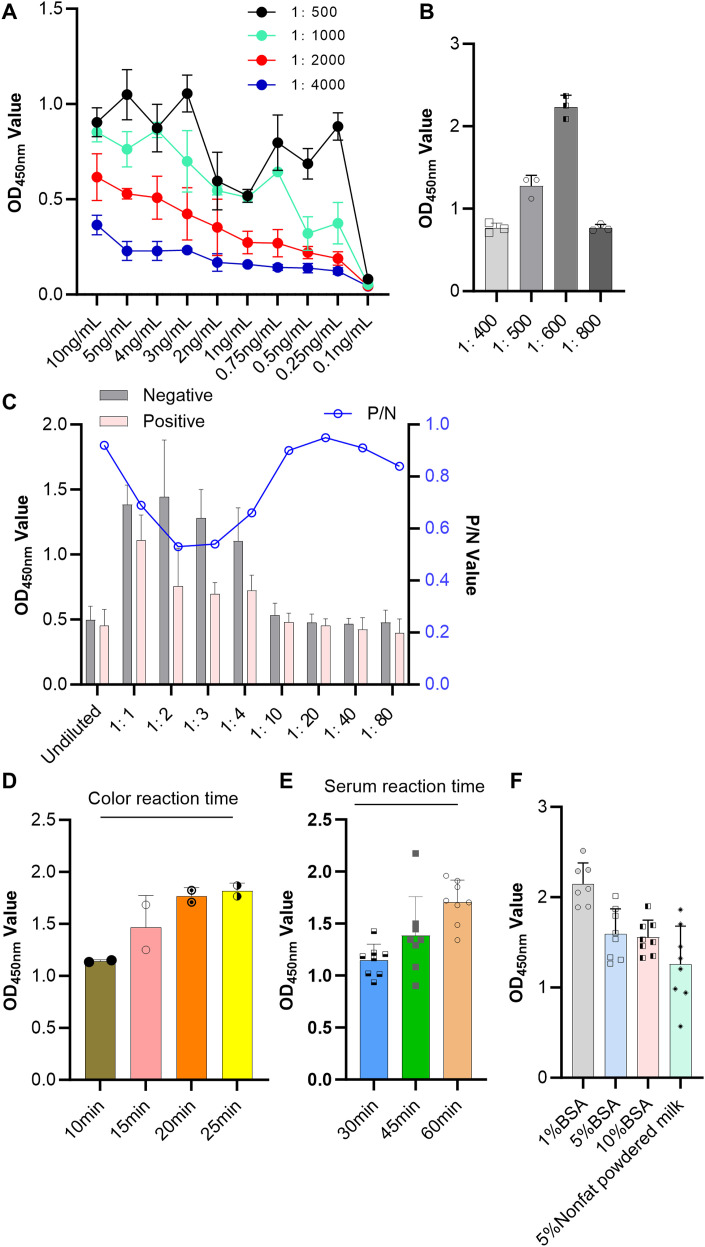
Screening of optimal dilutions for blocking ELISA.**(A)** Preliminary screening of protein-coating concentration and monoclonal antibody dilution. **(B)** Optimization of the optimal monoclonal antibody dilution at a protein-coating concentration of 3 ng/mL. **(C)** Screening of the optimal serum dilution under the conditions of a coating protein concentration of 3 ng/mL and a specific monoclonal antibody dilution of 1:600. **(D)** Screening results for different color development times. **(E)** Screening results for different serum reaction times.**(F)** Screening results for different blocking buffers.

To determine the optimal protein coating concentration, serum dilution, and monoclonal antibody dilution (mAb), a checkerboard titration method was used. First, the optimal dilutions of the coated protein and antibody were tested. As shown in **Figure 2A**, at an mAb dilution of 1:500, peak OD_450_ values were observed at protein coating concentrations of 5 and 3 ng/mL; however, neither OD_450_ value reached 1.5. To determine the optimal mAb dilution, the protein was coated at 3 ng/mL, and the mAb was serially diluted at ratios of 1:400, 1:500, 1:600, and 1:800 for testing. As shown in **Figure 2B**, the examination results indicated that the best reactivity was at an mAb dilution of 1:600, with an OD_450_ value of approximately 2.0. To identify the optimal serum dilution, the serum was serially diluted using the previously determined coating and mAb dilution concentrations. The optimal serum dilution was determined based on an OD450 value of approximately 1.5 in the negative control and the lowest positive/negative (P/N) ratio. As shown in **Figure 2C**, when the serum was diluted 1:2, the negative serum OD_450_ value was approximately 1.5-2.0, and the P/N ratio was the lowest among all tested dilutions.

To further optimize the assay conditions, the blocking buffer (1%, 5%, 10% BSA, and 5% non-fat milk), serum incubation time (30, 45, and 60 min), and chromogenic substrate incubation time (10, 15, and 20 min) were evaluated. Optimal serum and substrate incubation times were selected based on a negative control OD_450_ value of approximately 1.5, whereas the optimal blocking buffer was determined using a target negative control OD_450_ value of approximately 2.0. To minimize variability, the chromogenic reaction time was optimized. As illustrated in **Figure 2D**, a 15 min substrate incubation yielded a negative control OD_450_ of approximately 1.5. Subsequently, the serum incubation time was optimized, and a 45 min incubation resulted in a negative control OD_450_ value of approximately 1.5 (**Figure 2E**). Finally, the composition and concentration of the blocking buffer were optimized, with 1% BSA yielding an OD_450_ value of approximately 2.0 for the negative control (**Figure 2F**). In summary, the optimal conditions for the blocking ELISA were as follows: protein coating concentration of 3 ng/mL, blocking with 1% BSA, serum dilution of 1:2, incubation for 45 min, monoclonal antibody dilution of 1:600, and chromogenic substrate incubation for 15 min. The final optimized parameters are summarized in **Table 1**.

**Table 1:** Optimization of reaction conditions for blocking ELISA.

Item	Protein coating amount	Blocking buffer	Serum	HRP-labeled specific monoclonal antibody	Color reaction time
Optimal concentration	3 ng/mL	1% BSA	1∶2	1∶600	—
Reaction condition	4 °C/16 h	37 °C/1 h	37 °C/45 min	37 °C/1h	37 °C/15min

### 3.3 Determination of the cutoff value for the blocking ELISA

To determine the cutoff value, 40 serum samples confirmed to be negative for PPRV using the WOAH-recommended competitive ELISA were selected. These samples were subsequently tested using a blocking ELISA under optimized conditions. The percent inhibition (PI) was calculated according to the following formula: PI = [(OD_450_ of negative control serum – OD_450_ of test serum)/OD_450_ of negative control serum] × 100%. The mean PI ( $\bar{X}$) and SD were calculated, and the cutoff value for the blocking ELISA was defined as $\bar{X}$ + 3SD. The mean inhibition rate of the 40 serum samples was 9.92%, with an SD of 8.53%. Therefore, a test sample with a PI greater than 35.51% was considered to be positive.

### 3.4 Specificity, sensitivity, and repeatability of the blocking ELISA

**Figure 3:**
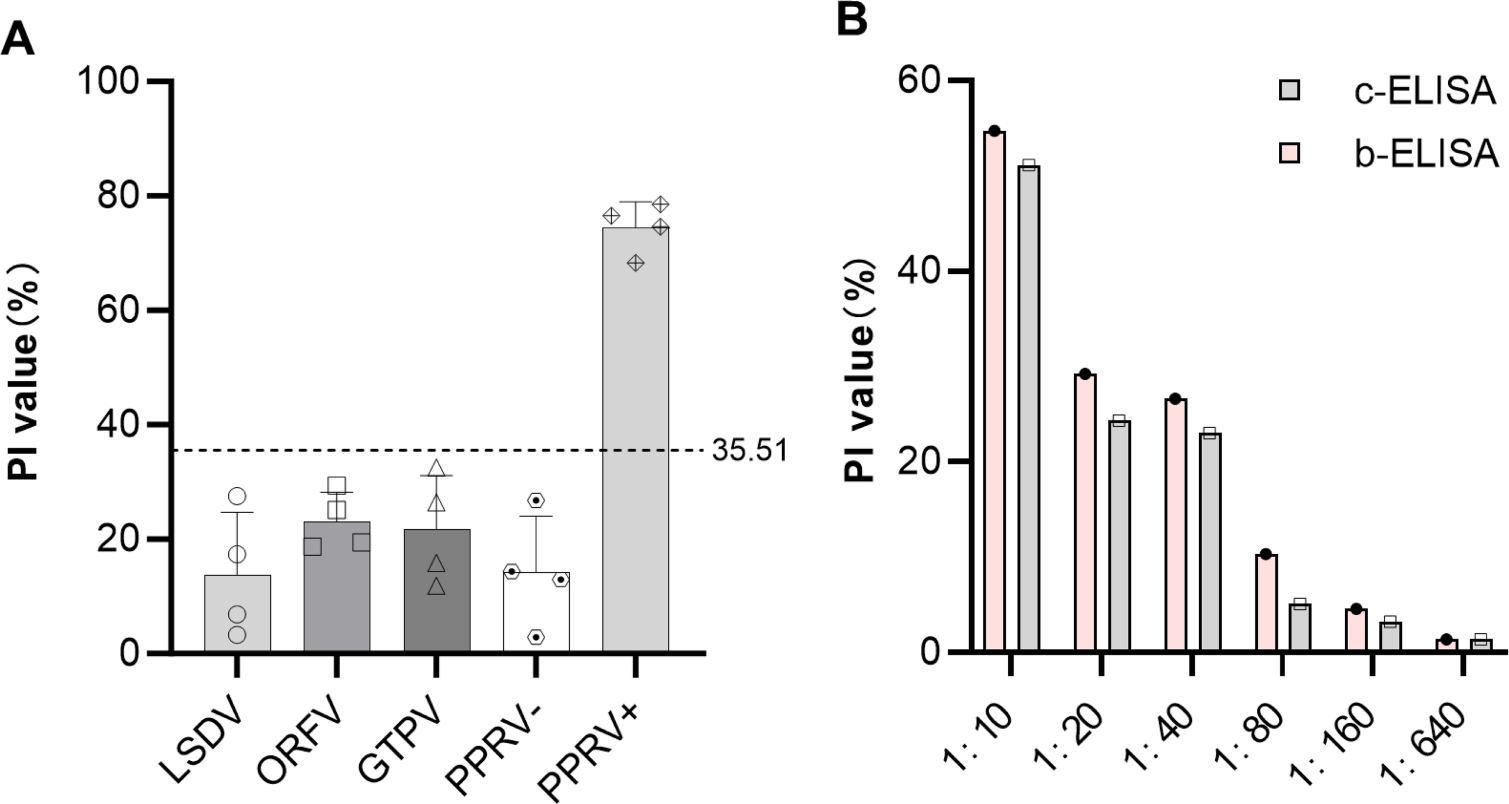
Determination of specificity and sensitivity of blocking ELISA. **(A)** Positive and negative serum reactions to LSDV, ORFV, GTPV, and PPRV.**(B)** Comparison of c-ELISA and b-ELISA results at the same positive serum dilution.

To assess the specificity of the established method, blocking ELISA was used to detect LSDV, ORFV, GTPV, and PPRV antibody-positive sera and PPRV-negative sera. As shown in **Figure 3A**, the inhibition rates for all sera, except the PPRV-positive serum, were below 35.51%, whereas the inhibition rate for the PPRV-positive serum was approximately 80%, indicating a good specificity.

To assess sensitivity, PPRV-positive serum was serially diluted and tested using both the established blocking ELISA and a commercial competitive ELISA kit. **Figure 3B** shows that our blocking ELISA had sensitivity comparable to or slightly higher than that of the commercial kit, detecting PPRV antibodies at a dilution of 1:10. As shown in **Table 2**, the repeatability results indicated that the coefficients of variation for intra-assay and inter-assay repeatability ranged from 8.097% to 11.895% and from 2.046% to 9.847%, respectively, both below 15%, confirming the reliable repeatability of the blocking ELISA.

**Table 2:** Repeatability test within and between batches.

Serum No.	Intra batch		Inter batch	
	$\bar{x}$ ± *s*	CV%	$\bar{x}$ ± *s*	CV%
1	62.9% ± 7.5%	11.895%	62.9% ± 1.7%	2.631%
2	35.4% ± 3.9%	8.097%	35.4% ± 1.6%	4.432%
3	25.4% ± 2.9%	11.477%	25.4% ± 2.5%	9.847%
4	19.9% ± 2.4%	11.796%	19.9% ± 0.41%	2.046%

### 3.5 Examination of the clinical sample testing using the blocking ELISA

To assess the concordance rate of the established blocking ELISA, the method was used in conjunction with the competitive ELISA kit developed by the Lanzhou Veterinary Research Institute to detect antibodies in 100 clinical sheep serum samples. The concordance rate (CR) was calculated using the following formula: CR (%) = (total number of samples – number of discrepant samples)/total number of samples × 100. As shown in **Table 3**, among the 100 samples tested, 93 showed consistent results between the two methods, whereas the remaining seven discrepant samples were all identified as negative upon further testing. Of the 100 clinical serum samples, the WOAH-recommended PPRV antibody competitive ELISA identified 20 as negative and 80 as positive samples. Both methods achieved a 100% detection rate for positive sera; however, the competitive ELISA for negative sera was not perfectly accurate. Because the concordance rate reported in **Table 3** was calculated assuming a 100% detection rate for competitive ELISA, the resulting concordance rate may be subject to error. When the concordance rate for the blocking ELISA was calculated independently using the formula, it was 95%, whereas that for the competitive ELISA was 98%. These results indicate good consistency between the two methods, suggesting that the established blocking ELISA can be effectively used for practical application.

**Table 3:** Comparison of c-ELISA and b-ELISA results.

c-ELISA	b-ELISA		Total	Coincidence rate
	Positive	Negative		
Positive	80	2	82	93%
Negative	5	13	18	
Total	85	15	100	

## 4 Discussion

In this study, a blocking ELISA was developed using a recombinant PPRV-2N protein and an HRP-labeled N protein mAb to detect PPRV antibodies, demonstrating high specificity, sensitivity, and repeatability. The developed ELISA method provides a reliable tool for PPRV antibody surveillance and offers significant value in evaluating vaccine immunity and supporting scientific efforts to prevent and control PPR.

Since its first report, PPR has continually expanded its reach to affected countries and species, resulting in annual global economic losses of up to several billion US dollars (Jones et al., 2016). To effectively control and ultimately eradicate PPR, efficient vaccination strategies and rapid and sensitive detection methods must be developed. Currently, numerous techniques are available for detecting animal viruses and antibodies, such as HI for avian influenza and Newcastle disease antibody titers, high-sensitivity fluorescent test strips for Brucella (Kong et al., 2021), and the RPA/CRISPR rapid detection method for the H5 subtype of avian influenza virus (Zhou et al., 2024). These methods facilitate the rapid and accurate on-site detection of viruses or antibodies, thereby contributing to the prevention and control of animal disease. PPRV infection or vaccination prompts the production of protective antibodies in the host (Saravanan et al., 2010). Developing a rapid, sensitive, and specific method for monitoring antibody levels in vaccinated animals and conducting sero-epidemiological surveys is vital for evaluating vaccine efficacy and providing a scientific basis for guiding epidemic prevention and planning eradication efforts (Singh, 2011). In China, the current methods for detecting PPRV antibodies include VNT, HI, CIE, ELISA, SPR biosensors, colloidal gold, and time-resolved fluorescence immunoassays. Competitive ELISA is the standard detection method currently recommended by WOAH. Each technique has its advantages and disadvantages. Some studies have suggested that traditional competitive ELISA procedures can be relatively cumbersome and time-consuming, whereas blocking ELISA is simpler to perform, offers higher accuracy and repeatability, and is applicable across a broader range of species (Saliki et al., 1993; Lelisa et al., 2022). Blocking ELISA, which operates similarly to competitive ELISA, relies on highly specific antigen–antibody interactions, remains unaffected by the hemagglutination properties of the virus, provides high sensitivity, and delivers stable and accurate results. It is objective, cost-effective, and suitable for large-scale screening and field application. In this study, a recombinant protein, 2N, targeting the dominant antigenic epitopes of the PPRV N protein, was successfully constructed. Using this protein as the coating antigen in combination with an HRP-labeled monoclonal antibody against the PPRV N protein, a blocking ELISA was established to detect PPRV antibodies in the sera of small ruminants. Compared with traditional ELISA methods based on the whole virus or full-length N protein, the blocking ELISA developed in this study offers the following advantages: first, the recombinant protein 2N expressed in a prokaryotic system is relatively simple to prepare, with good batch-to-batch consistency and ease of standardization; second, the competitive reaction mode of blocking ELISA effectively reduces background noise compared to indirect ELISA, thereby enhancing detection specificity; and third, this method does not rely on live virus antigens, offering high biosafety and suitability for routine laboratory applications.

The PPRV N protein is the most abundant, highly conserved, and antigenically stable protein in the virus, making it a crucial target for developing PPRV antigen- and antibody-detection methods. For example, Xu et al. established a rapid RAA-CRISPR Cas12a detection method for PPRV based on the N protein (Xu et al., 2024), Dong et al. developed a colloidal gold test strip for PPRV antibody detection using the N protein (Dong et al., 2023), and Choi et al. established a c-ELISA detection method for PPRV using the N protein (Choi et al., 2005). The H protein is another target for establishing PPRV detection methods. For instance, Bodjo et al. developed a blocking ELISA for PPRV antibody detection using monoclonal antibodies against the H protein (Bodjo et al., 2018), and Hu et al. established a competitive ELISA for detecting neutralizing antibodies targeting the H protein (Hu et al., 2025). Currently, few commercial kits for PPRV serum detection are available, and most are competitive ELISA kits, such as the ID Screen PPR Competition kit. Here, the blocking ELISA showed good specificity, with no cross-reactivity with sera from animals positive for related pathogens, including LSDV, GTPV, and ORFV. It could detect positive serum diluted up to 1:10, with intra-assay and inter-assay CVs below 15%, indicating that the monoclonal antibody specifically recognized the PPRV-N protein and provided a reliable basis for accurately identifying PPRV infection in cases of co-infection. In clinical validation, the overall concordance rate was 95%, indicating a high consistency with mainstream methods. For individual samples with inconsistent results, the discrepancies may arise from two factors: first, differences in the coating antigens and competing or blocking antibodies used by the two methods, resulting in variable reactivity with certain sera; second, inherent differences in detection principles (blocking vs. competitive ELISA) lead to different balances between sensitivity and specificity. These inconsistencies suggest that the established method can effectively supplement existing detection systems by providing an alternative perspective for assessing the immune status of animals.

However, this study had several limitations. First, the correlation between the antibody levels detected by this blocking ELISA and neutralizing antibody titers remains to be elucidated. Future studies should include parallel comparisons with neutralization tests to assess the utility of this method in accurately assessing vaccine-induced immune responses. Second, as antibodies targeting the N protein are commonly elicited by both natural infection and vaccination, this method does not permit differentiation between infected and vaccinated animals. To address this limitation, additional serological assays based on non-structural proteins are required. Furthermore, further validation of assay specificity is warranted, including additional positive sera against related pathogens and samples that are negative for PPRV but positive for other pathogens. Similarly, the clinical sample size should be expanded to encompass a broader range of immune statuses and breeding environments, thereby enabling robust statistical analyses to assess the agreement with a commercial reference kit. Finally, sensitivity data should be supplemented by testing serially diluted positive sera in parallel with a commercial kit or neutralization test, enabling the calculation of relative sensitivity.

## 5 Conclusion

In summary, this study successfully established a blocking ELISA for the detection of PPRV antibodies based on a recombinantly expressed tandem protein encompassing the dominant antigenic epitopes of the PPRV N protein. Using overlap PCR, a prokaryotic expression plasmid (pET28a-PPRV-2N) was constructed, and the recombinant protein PPRV-2N was expressed and purified. The method demonstrated high sensitivity, detecting positive serum at a dilution of 1:10, with intra-assay and inter-assay coefficients of variation below 15%. Specificity analysis revealed no cross-reactivity with sera positive for LSDV, GTPV, or ORFV, indicating excellent specificity. Furthermore, clinical sample testing yielded a 93% concordance rate with a commercial detection kit, confirming the reliability of the method. These findings indicate that the blocking ELISA developed in this study is a sensitive, specific, and reproducible tool for sero-monitoring and disease control in small ruminants. This approach holds significant potential for application in epidemiological surveillance and outbreak monitoring, thereby advancing efforts to eradicate PPR.

## Data Availability

The original contributions presented in the study are included in the article/**Supplementary Material**. Further inquiries can be directed to the corresponding authors.

## Supplementary material

Supplementary Figure 1 Dominant epitope prediction analyses of the N protein of virulent and attenuated PPRV. **(A)** Schematic representation and dominant epitope prediction of full-length N of attenuated PPRV. B-cell epitopes were predicted using the online IEDB analysis resource (http://tools.iedb.org/bcell/). **(B)** Schematic representation and dominant epitope prediction of full-length N of virulent PPRV. B-cell epitopes were predicted using the online IEDB analysis resource (http://tools.iedb.org/bcell/). **(C)** Schematic representation and dominant epitope prediction of 2N of PPRV. B-cell epitopes were predicted using the online IEDB analysis resource (http://tools.iedb.org/bcell/).
