# Supplementary figures and images for: Establishment of a blocking enzyme-linked immunosorbent assay based on tandem expression of dominant antigenic epitopes of the nucleocapsid protein from attenuated and virulent peste des petits ruminants virus
Source: Front Cell Infect Microbiol. 2026 Jun 3;16:1841234. doi: 10.3389/fcimb.2026.1841234 (PMC13272494; doi:10.3389/fcimb.2026.1841234)

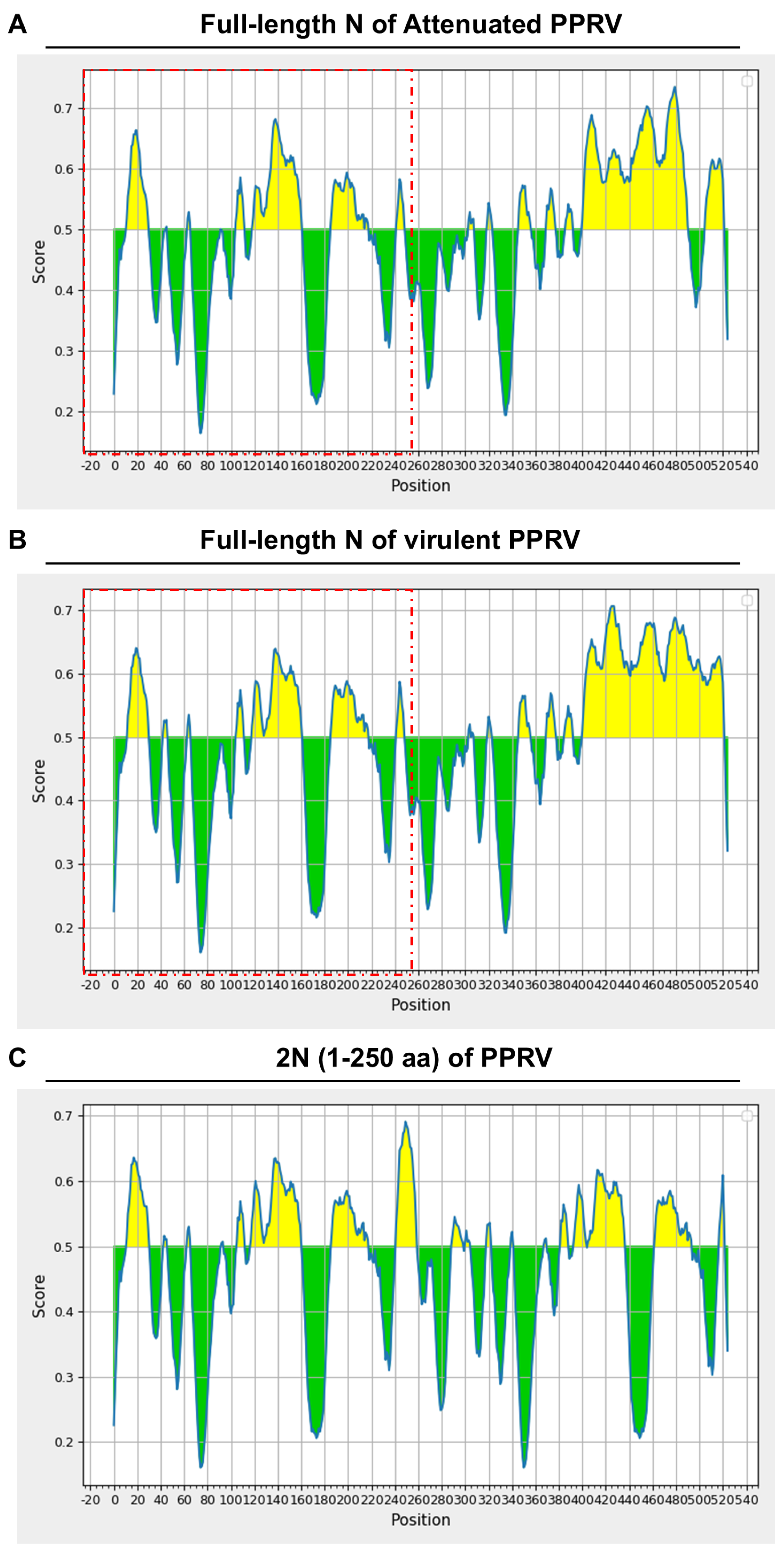

Supplement: Supplementary Figure 1 — Dominant epitope prediction analyses of the N protein of virulent and attenuated PPRV. (A) Schematic representation and dominant epitope prediction of full-length N of attenuated PPRV. B-cell epitopes were predicted using the online IEDB analysis resource (http://tools.iedb.org/bcell/). (B) Schematic representation and dominant epitope prediction of full-length N of virulent PPRV. B-cell epitopes were predicted using the online IEDB analysis resource (http://tools.iedb.org/bcell/). (C) Schematic representation and dominant epitope prediction of 2N of PPRV. B-cell epitopes were predicted using the online IEDB analysis resource (http://tools.iedb.org/bcell/). [file Image1.tif]
